# Prevalence, Risk Factors, Antimicrobial Resistance and Molecular Characterization of *Salmonella* in Northeast Tunisia Broiler Flocks

**DOI:** 10.3390/vetsci9010012

**Published:** 2021-12-30

**Authors:** Walid Oueslati, Mohamed Ridha Rjeibi, Hayet Benyedem, Aymen Mamlouk, Fatma Souissi, Rachid Selmi, Abdelfettah Ettriqui

**Affiliations:** 1 Laboratory of Management of Animal Production’s Health and Quality, National School of Veterinary Medicine of Sidi Thabet, University Manouba, La Manouba 2010, Tunisia; souissifatma83@yahoo.fr (F.S.); abdelfettah.ettriqui@enmv.uma.tn (A.E.); 2 Department of Animal Production, National Agronomic Institute, University Carthage, Carthage 1054, Tunisia; 3 Laboratory of Parasitology, National School of Veterinary Medicine of Sidi Thabet, University Manouba, La Manouba 2010, Tunisia; medridharjeibi@yahoo.fr (M.R.R.); benyedemhayet@gmail.com (H.B.); 4 Laboratory of Parasitology, Veterinary Research Institute, University de Tunis El Manar, Tunis 1068, Tunisia; 5 Laboratory of Microbiology, National School of Veterinary Medicine of Sidi Thabet, University Manouba, La Manouba 2010, Tunisia; aymen.mamlouk@enmv.uma.tn (A.M.); selmiveto1983@gmail.com (R.S.)

**Keywords:** *Salmonella*, broiler flocks, risk factors, MDR strains, ESBL, Tunisia

## Abstract

This study was conducted in northeastern Tunisia to estimate both the prevalence and the risk factors of *Salmonella* in broiler flocks as well as to characterize the isolated multidrug-resistant (MDR) *Salmonella* strains. In the present study, a total number of 124 farms were sampled; *Salmonella* isolates were identified by the alternative technique *VIDAS Easy Salmonella*. The susceptibility of *Salmonella* isolates was assessed against 21 antimicrobials using the disk diffusion method on Mueller–Hinton agar using antimicrobial discs. Some antimicrobial resistance genes were identified using PCR. The prevalence rate of *Salmonella* infection, in the sampled farms, was estimated at 19.9% (64/322). Moreover, a total number of 13 different serotypes were identified. High rate of resistance was identified against nalidixic acid (82.85%), amoxicillin (81.25%), streptomycin (75%), and ciprofloxacin (75%). Alarming level of resistance to ertapenem (12.5%) was noticed. A total of 87.5% (56/64) of isolated strains were recognized as MDR. Three MDR strains were extended-spectrum β-lactamases (ESBL)-producers and three MDR strains were cephalosporinase-producers. The *bla_CTX__-M_* gene was amplified in all the three ESBL strains. The *qnrB* gene was not amplified in fluoroquinolones-resistant strains. The *tetA* and *tetB* genes were amplified in 5% (2/40) and 2.5% (1/40) of tetracycline-resistant strains, respectively. The *dfrA1* gene was amplified in five of the 20 trimethoprim-resistant strains. The *mcr-1, mcr-2, mcr-3, mcr-4,* and *mcr-5* genes were not amplified in any of the phenotypically colistin-resistant strains. In terms of integrase genes int1 and int2, only gene class 2 was amplified in 11% (7/64) of analyzed strains. Risk factors, such as the poor level of cleaning and disinfection, the lack of antimicrobial treatment at the start of the breeding, and a crawl space duration lower than 15 days, were associated with high *Salmonella* infection in birds. These data should be considered when preparing salmonellosis control programs in Tunisian broiler flocks.

## 1. Introduction

According to numerous surveys, undercooked poultry meat is often reported as responsible for non-typhoid *Salmonella* gastroenteritis outbreaks with *Salmonella* Typhimurium and *Salmonella* Enteritidis being the most frequently isolated serotypes [1,2,3]. The Centers for Disease Control and Prevention (CDC) estimated that *Salmonella* bacteria cause, yearly, 1.35 million infections, 26,500 hospitalizations, and 420 deaths in the United States [4]. Poultry products are the source of most of these cases [5]. In Europe, since 2014, *Salmonella* spp. has been the second-highest bacterial agent, after *Campylobacter*, causing gastroenteritis in humans [6]. In Sub-Saharan Africa, non-typhoid salmonellosis, mostly caused by *S*. Typhimurium and *S*. Enteritidis, is a major public health problem [2]. In Tunisia, *Salmonella* food-borne infections are an emerging public health problem [7], with *S*. Kentucky and *S.* Anatum being the most identified serotypes in poultry meat [8].

The breeding period represents a critical stage for the development of *Salmonella* in broiler flocks [9]. Identification of the risk factors associated with broilers’ infection with these bacteria is therefore essential in order to prevent its occurrence [9]. In Tunisia, a national program for the control of zoonotic *Salmonella* infections is performed for only poultry reproductive herds. This program aims to reduce the prevalence of these infections by setting up surveillance. To prevent the spread of the infection outside the poultry farm, all poultry are euthanized when an infection is confirmed in any poultry reproductive farm.

Non-typhoid *Salmonella* gastroenteritis is expressed by digestive signs (diarrhea, vomiting, and abdominal pain) associated with fever and depression. The first symptom appears approximately 12 to 24 h after ingestion of the contaminated food. The acute phase lasts approximately 24 to 48 h. Symptoms resolve spontaneously, and symptomatic treatment is generally sufficient [10]. In children, elderly, and immune-compromised persons, digestive infections can progress to sepsis and meningitis, leading to death. Antimicrobial therapy is therefore prescribed to particularly sensitive persons [11]. Uncontrolled use of antimicrobials in poultry farming leads to the selection of multidrug-resistant *Salmonella* strains [12]. The alarming increase of antimicrobial resistance is another aspect of the public health concern of *Salmonella* infection [12]. It was shown that a proportion of multidrug-resistant *Salmonella* found in humans are of animal origin and have acquired their resistance genes in breeding before being transmitted to humans through food [13].

In Tunisia, data is still lacking, and only one study of Bichiou et al. (2010) [14] is available regarding the infection of broiler chickens by non-typhoid *Salmonella*. The present study was conducted in northeastern Tunisia to estimate both the prevalence and the risk factors of *Salmonella* in broiler flocks as well as to characterize the isolated multidrug-resistant (MDR) *Salmonella* strains.

## 2. Materials and Methods

### 2.1. Choice of Breeding Sites

The northeast region of Tunisia is one of the main economic regions that covers seven governorates (Tunis, Ariana, Ben Arous, Manouba, Bizerte, Nabeul, and Zaghouan) and represents the most populated region, with 37% of the Tunisian population and a high demographic density (344 inhabitants/km^2^ compared to the mean Tunisian density: 69 inhabitants/km^2^). Northeast Tunisia is characterized by an intense commercial poultry farming, in particular broilers, totaling 1830 broiler farms (41% of broiler Tunisian farms) producing 49 million chickens/year (45% of national production in 2019) [15].

### 2.2. Sampling

**Figure 1:**
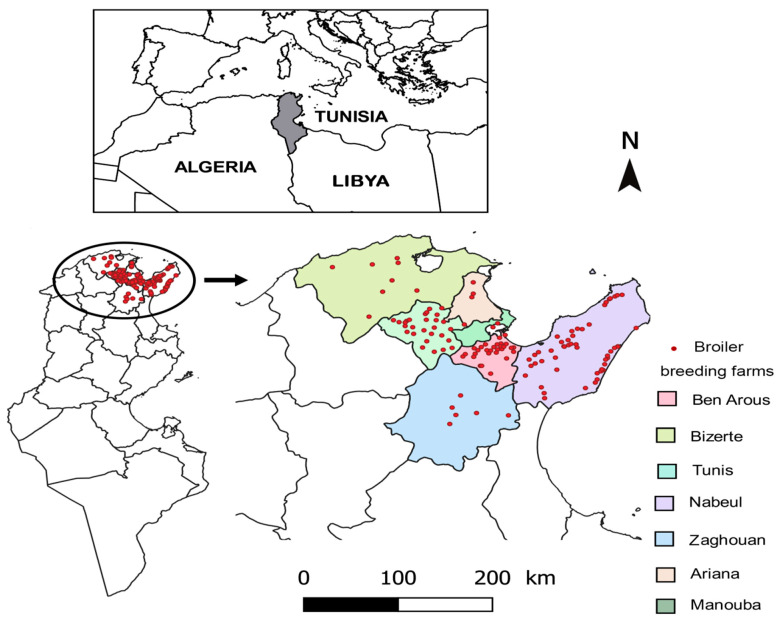
Geographic localization of the studied broiler breeding farms.

The present study was conducted between September 2019 and August 2020 on broiler chicken farms located in Northeast Tunisia (Figure 1).

The required number of farms (n) was estimated using the formula of Thrusfield [16] for a 95% confidence interval (95% CI): n = 1.96 P_exp_ (1 − P_exp_)/D^2^, where P_exp_: expected prevalence which was estimated to 17% [14] and D: the precision that was fixed to 5%.

A total number of 124 broiler chicken farms were included in the present study located in Northeast Tunisia (Figure 1). Visited farms were selected according to the willingness of the owners to participate in the current study. In each farm, at least 50% of the poultry house-buildings were randomly included, totaling 322 breeding units. The number of chickens per poultry house-building varied between 10,000 and 25,000. In order to estimate the prevalence of *Salmonella* presence in the fecal samples of broiler chicken, ten pools of five fresh droppings were collected in sterile flasks per building. Each poultry flock was sampled once during the production cycle when chickens were two to four weeks old. A total number of 224 and 98 samples were collected in the hot season and the cold season, respectively. The number of samples was lower during the cold season because the number of house-buildings occupied by poultry is lower than during the hot season. In fact, due to the high cost of house-building heating, the production cost is higher during the cold season. Information about the main risk factors, including location, environment, infrastructure, hygiene and biosecurity standards, animal welfare, and health control were collected. The risk factors were typed by questionnaire. The risk factors were assessed based on compliance with good practice and meeting the five animal needs, such as need for a suitable environment, need for a suitable diet, need to be able to exhibit normal behavior patterns, need to be housed with, or apart from, other animals, and need to be protected from pain, suffering, injury, and disease [17].

### 2.3. Salmonella spp. Screening

All samples of fresh droppings were screened for *Salmonella* by the alternative technique VIDAS Easy *Salmonella* (bioMérieux SA, Lyon, France). This technique is based on the detection of specific *Salmonella* proteins by immune-fluorescence after primary and secondary enrichment followed by lysis of the bacteria by heating at 100 °C. Primary enrichment consists of incubating, at 37 °C for 22 h, a mixture of 25 g of fresh droppings samples and 225 mL of Buffered Peptone Water (Biokar Diagnostics, Beauvais, France). After incubation, 100 μL of the mixture was transferred into 10 mL of *Salmonella* Xpress 2 (SX2) broth (bioMérieux SA) and incubated at 41.5 °C for 24 h. One milliliter of the mixture was boiled for 15 min, then cooled to room temperature. To detect *Salmonella* target proteins, a volume of 0.5 mL of the already-cooled mixture was transferred to an SLM chip and deposited at VIDAS (bioMérieux SA). The unheated SX2 broth is used for the isolation of *Salmonella*, from positive samples, on selective agar (XLD and SS) (Biokar Diagnostics, Beauvais, France). Confirmation is performed by urease test followed by API 20E system test (bioMérieux SA) according to the reference technique ISO 6579, 2017 [18].

### 2.4. Salmonella Strains Serotyping

The rapid agglutination on the slide with specific immune sera against the O, H, and Vi antigens of *Salmonella* (BioRad, Paris, France) was the serotyping technique performed in the present study at Pasteur Institute of Tunis (Tunis, Tunisia). The identification of serotypes was based on the White–Kauffmann–Le Minor scheme [19].

### 2.5. Molecular Study 

The extraction of *Salmonella* genomic DNA was carried out from the colonies present in the unheated SX2 broth (bioMérieux SA) using the ONE-4-ALL GENOMIC DNA Mini kit. Preps (Bio Basic, Markham, ON, Canada). After centrifugation of the SX2 broth for 1 min at 10,000 rpm, the pellet was mixed with washing solution and proteinase K (20 mg/mL). A second centrifugation of the mixture (already heated to 56 °C for one hour) was performed at 12,000 rpm for 2 min. After washing the pellet, the DNA was eluted by incubation at room temperature for 2 min, followed by centrifugation at 9000 rpm for 2 min, then stored at −20 °C until used.

Molecular screening for *Salmonella* was performed by PCR targeting a 1 kb DNA fragment [20], using specific primers (F5′ACCACGCTCTTTCGTCTGG3′ and R-5GAACTGACTACGTAGACGCTC3′) [20]. In addition, 12 virulence genes (Table 1) [21,22,23] were screened in *Salmonella* isolates. PCR was performed with an Esco Swift Max Pro thermal cycler (Horsham, PA, USA) in a total volume of 25 µL containing 1 U Taq Polymerase (Bio Basic, Markham, ON, Canada), 1 × PCR buffer (5 mM KCl Tris-HCl, pH 8.5), 1.5 mM MgCl_2_, 0.1 mM dNTP (Bio Basic, Markham, ON, Canada), 1 µM forward and reverse primer (Bio Basic, Markham, ON, Canada) and 1 µL DNA. Denaturation was carried out first at 95 °C for 10 min followed by 35 cycles (denaturation at 95 °C for 1 min, hybridization for 1 min at temperatures depending on the target gene and elongation at 72 °C for 1 min) and a final extension at 72 °C for 10 min. A total number of 12 primers were used for the detection of the target genes. The amplicons were visualized in an agarose gel. Negative (sterile distilled water) and positive (*Salmonella* strains isolated in a previous published and unpublished studies [24]) controls were added in each PCR cycle.

### 2.6. Antimicrobial Susceptibility Testing and Identification of Antimicrobial Resistance Genes

Diffusion method on Mueller–Hinton agar using antimicrobial discs (Bio-Rad, Marne-La-Coquette, France) was performed to test the resistance of *Salmonella* strains to 21 antimicrobials. Results were interpreted as recommended by the Antibiogram Committee of the French Society for Microbiology—Veterinary Antibiograms [28]. Colistin resistance was detected by Colispot test [29]. The presence of an inhibition zone after an application of a single drop of 8 mg/L colistin solution on a previously inoculated Mueller–Hinton agar indicated that *Salmonella* isolates were susceptible to colistin [29]. A screening test of resistance genes beta-lactams (*bla_TEM_, bla_NDM1_* and *bla_CTX-M_*), fluoroquinolones (*qnrB*), tetracycline (*tet(A)* and *tet(B)*), trimethoprim(*dfrA1*), colistin (*mcr-1 to mcr-5*), and class 1 and 2 integrons was performed by PCRs as described above (Table 2) [26,30,31,32,33,34,35,36].

### 2.7. Statistical Analysis

Eight factors were selected according to known *Salmonella* infection risk factors in poultry farms. In addition, 21 variables that represent used antimicrobials were included in the statistical analysis. Data were analyzed with XLSTAT^®^ (Addinsoft, New York, NY, USA) and SPSS (Version 23.0, IBM Corp; New York, NY, USA). The Pearson chi-square test aims to assess relation between the data collected on poultry farms and the infection of the broiler flock with *Salmonella* at a threshold value of 5%. Logistic regression was used to assess the effect of the parameters studied on *Salmonella* infection prevalence; both odds ratios and 95% confidence intervals were estimated. The retrospective likelihood ratio selection method was used, and a *p*-value cut-off of 0.1 was considered for selecting the risk factors to be included in the model. The goodness of fit was assessed using Hosmer and Lemeshow χ2 test [38]. The area under curve (AUC) of the receiver operating characteristic (ROC) was plotted as an estimation of the predictive ability of the model. Principal component analysis (PCA) was performed to investigate correlations between the isolated strains and the antimicrobial resistance profiles.

## 3. Results

### 3.1. Prevalence of Salmonella spp. in Broiler

**Figure 2:**
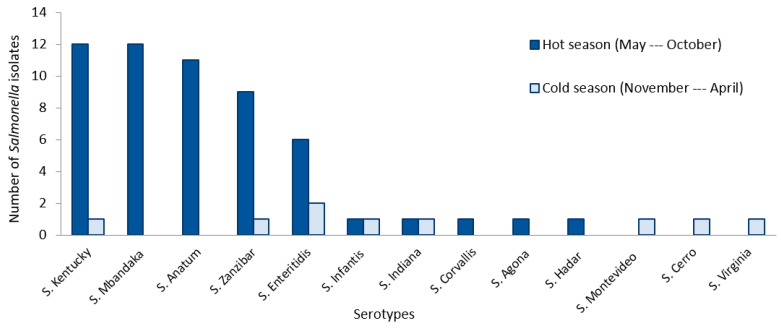
Seasonal distribution of *Salmonella* spp. serotypes in the studied Tunisian broiler breeding farms.

The prevalence of *Salmonella* presence in the fecal samples of broiler chicken was estimated at 19.9% (64/322). The 64 isolates were positive to PCR using *Salmonella* specific primers. Prevalence of *Salmonella* broiler infection was significantly higher in hot season (24.6%; 55/224) compared to cold season (9.2%; 9/98) (*p* < 0.05) (Table 3, Figure 2). A total number of 13 serotypes were identified, namely, *S*. Kentucky (20.3%;13/64), *S.* Mbandaka (18.7%; 12/64), *S*. Anatum (17.1%; 11/64), *S*. Zanzibar (15.6%; 10/64), *S.* Enteritidis (12.5%; 8/64), *S.* Infantis (3.1%; 2/64), *S.* Indiana (3.1%; 2/64), *S.* Corvallis (1.6%; 1/64), *S.* Agona (1.6%; 1/64), *S.* Hadar (1.6%; 1/64), *S.* Montevideo (1.6%; 1/64), *S.* Cerro (1.6%; 1/64), and *S.* Virginia (1.6%; 1/64) (Figure 2).

### 3.2. Risk Factors Associated with the Presence of Salmonella in the Fecal Samples of Broiler Chicken

The logistic regression based on univariate analysis used to evaluate the impact of eight factors selected according to known *Salmonella* infection in poultry farms revealed that broiler infection with *Salmonella* spp. was significantly related to at least six risk factors, namely, hot season (T ≥ 20 °C), duration of crawl space lower than 15 days, absence of treatment with an antimicrobial at the start, wet litter, no cleaning and disinfection around the breeding units, and number of chicks higher than 25/m^2^ (*p* < 0.05) (Table 3).

Seasonal fluctuation of broiler contamination by *Salmonella* showed a significant difference between cold and hot season that was characterized in Northeast Tunisia by an average ambient temperature of ≥20 °C (OR = 3.218; 95% CI = 1.520–6.813) (Table 3). Unlike other serotypes, *S*. Kentucky, *S.* Mbandaka, *S*. Anatum, *S*. Zanzibar, and *S.* Enteritidis were present during the whole hot season. They showed a prevalence peak during hot season (78.1%; 50/64) (*p* < 0.05) (Figure 2).

Besides the effect of the season, the presence of *Salmonella* spp. in broiler farm buildings is significantly influenced by a lack of zootechnical and biosecurity standards, as well as the lack of compliance with animal welfare rules: duration of crawl space lower than 15 days (OR = 6.590; 95% CI = 3.290–13.200), absence of treatment with an antimicrobial at the start (OR = 6.420; 95% CI = 3.270–12.610), wet litter (OR = 3.520; 95% CI = 1.850–6.680), no cleaning and disinfection around the breeding units (OR = 2.810; 95% CI = 1.600–4.950), and number of chicks higher than 25/m^2^ (OR = 2.720; 95% CI = 1.480–4.990) (Table 3).

The eight factors selected according to recognized *Salmonella* infection risk factors in poultry farms were included in multivariate logistic regression analysis. Using backward likelihood ratio selection method, three factors were left in the final model, namely, no cleaning and disinfection around the breeding units, no treatment with an antimicrobial at the start, and duration of crawl space lower than 15 days (Table 3).

**Figure 3:**
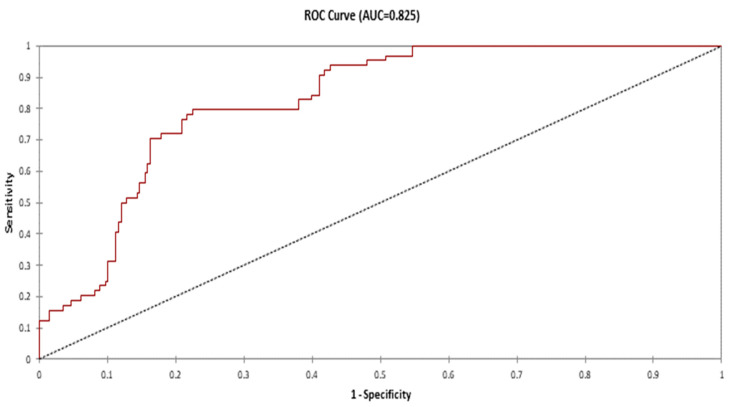
Receiver operating characteristic (ROC) curve plotted to measure the predictive ability of the model. Area under the curve (AUC) = 0.825.

*Salmonella* broiler flock contamination risk was significantly higher in breeding units where no cleaning and disinfection around the breeding units were performed (OR = 8.642; 95% CI = 1.770–42.196), no treatment with an antimicrobial at the start was performed (OR = 4.675; 95% CI = 1.720–12.703), and duration of crawl space was lower than 15 days (OR = 3.562; 95% CI = 1.436–8.835). The goodness of fit of the model was assessed using Hosmer and Lemeshow χ2 test. The area under the ROC curve (AUC) was estimated to 0.825 (Figure 3), allowing qualifying the model as good. This means that the obtained results regarding risk factors are reliable.

### 3.3. Prevalence of Virulence Genes

**Figure 4:**
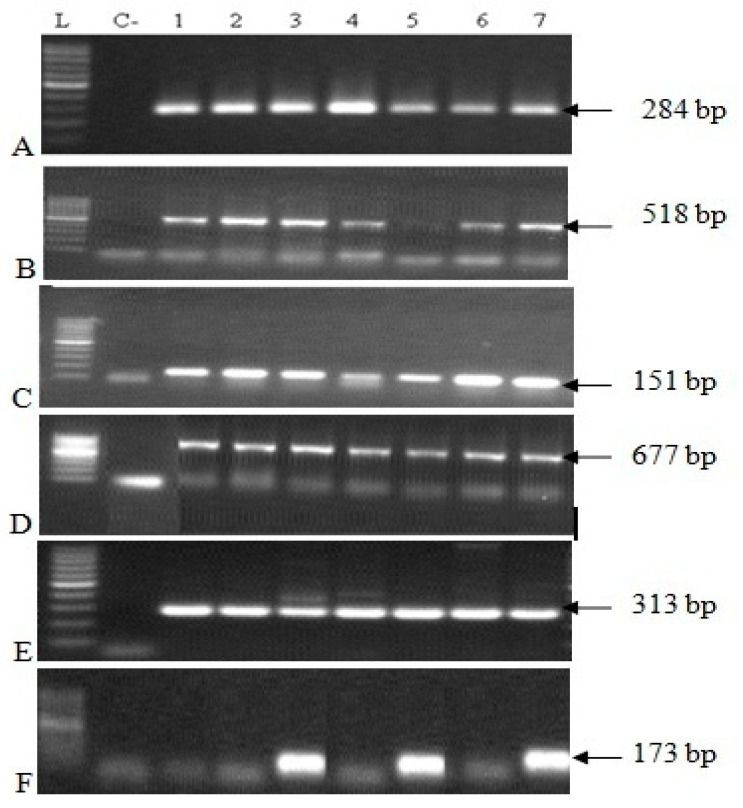
Agarose gel electrophoresis of *invA*, *gipA*, *pagK*, *mgtC*, *sirA*, and *Hli* genes amplicons. L: 100 bp ladder; C-: Negative control; 7: Positive control; Lanes 1 to 6: positive samples. (**A**): *invA*-positive samples; (**B**): *gipA*-positive samples; (**C**): *pagK*-positive samples; (**D**): *mgtC*-positive samples; (**E**): *sirA*-positive samples; (**F**): *Hli*-positive samples.

Four different virulotypes were found in the 64 analyzed strains of *Salmonella* (Table 4). These virulotypes were *invA-gipA-pagK-mgtC-sirA* (51.60%; 33/64), *invA-gipA-pagK-mgtC-sirA-Hli* (29.70%; 19/64), *invA-pagK-mgtC-sirA* (14%; 9/64), and *invA-pagK-mgtC-sirA-Hli* (4.70%; 3/64). All *Salmonella* (64strains) were positive for the genes *invA* (host cell invasion)*, pagK* (biofilm formation)*, mgtC* (intracellular survival), *and sirA* (control enteropathogenic virulence functions) and negative for the virulence genes *spvC, trhH, SEN1417, sipA, sipD,* and *sopD* (Figure 4, Table 4).

### 3.4. Antimicrobial Susceptibility Testing

**Figure 5:**
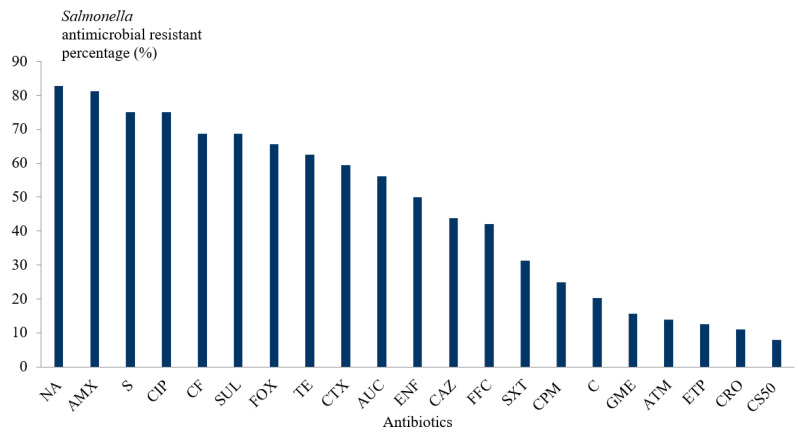
Percentage of antimicrobial-resistant *Salmonella* spp. isolated strains in Tunisian broiler breeding farms.AMX: amoxicillin, AUC: amoxicillin + clavulanic acid, SF: cefalotin, FOX: cefoxitin, CAZ: ceftazidim, CTX: cefotaxim, CRO: ceftriaxon, CPM: cefepim, ATM: aztreonam, ETP: ertapenem, GME: gentamicin, S: streptomycin, CS50: colistin, NA: nalidixic acid, ENF: enrofloxacin, CIP: ciprofloxacin, FFC: florfenicol, C: chloramphenicol, TE: tetracycline, SUL: sulfamides, SXT: trimethoprim-sulfamethoxazole.

High resistance rates were detected for nalidixic acid (82.85%; 53/64), amoxicillin (81.25%; 52/64), streptomycin (75%; 48/64), and ciprofloxacin (75%; 48/64). Alarming level of resistance to ertapenem (12.5%; 8/64) was noticed. However, resistances to colistin (7.85%; 5/64), ceftriaxon (11%; 7/64), aztreonam (14%; 9/64), gentamicin (15.6%; 10/64), and trimethoprim-sulfamethoxazole (31.25%; 20/64) were significantly lower than the other antimicrobials (*p* < 0.05) (Figure 5, Table 5).

All of the strains were resistant to at least two antimicrobials, and 87.5% (56/64) of strains were multidrug-resistant (MDR). The MDR strains were selected based on resistance to over three classes of antimicrobials.

**Figure 6:**
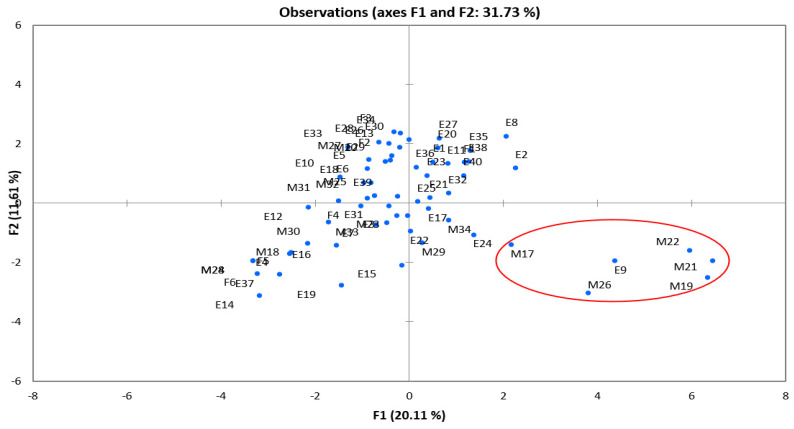
Principal component analysis (PCA) used to investigate correlations between the isolated strains and the antimicrobial resistance profiles. Red circle: outline of strains having distinct profiles from the others.

The principal component analysis (PCA) used to investigate correlations between the isolated strains and the antimicrobial resistance profiles revealed that six MDR strains (M17, M19, M21, M22, M26, and E9) had distinct profiles from the others (Figure 6). Three MDR strains (*S*. Anatum (M17, M22, and M26)) were extended-spectrum β-lactamase (ESBL)-producers, and three MDR strains (*S*. Anatum (M19, M21) and *S*. Corvallis (E9)) were cephalosporinase-producers (Table 5). The ESBL strains were selected based on resistance to amoxicillin, cephalosporines 1, 2, 3, 4 generations (except cefoxitin)and aztreonam, and sensitivity to the association (amoxicillin+ clavulanic acid) and ertapenem [28,39,40] In addition, ESBL strains can be characterized by the observation of a “champagne cork” synergy between the amoxicillin + clavulanic acid disc and a C3G/C4G disc or an aztreonam disc [28]. The AmpC strains were selected based on resistance to amoxicillin, the association (amoxicillin+ clavulanic acid), cephalosporines 1, 2, 3, 4 generations, and aztreonam [41].

### 3.5. Prevalence of Antimicrobial Resistance Genes

The *bla_CTX-M_* gene was identified in all the three ESBL strains. The *qnrB* gene was not identified in fluoroquinolone (enrofloxacine and ciprofloxacine)-resistant strains. The *tetA* and *tetB* genes were identified in 5% (2/40) and 2.5% (1/40), respectively, of tetracycline-resistant strains. The *dfrA1* gene was identified in five of the 20 trimethoprim-resistant strains. The genes *mcr-1 to mcr-5* were not identified in any of the five colistin-resistant strains. Integrase gene (int2) was identified in only 11% (7/64) of the *Salmonella* spp. strains.

## 4. Discussion

The results of our study showed that broiler infection prevalence with *Salmonella* spp. was 19.9% (64/322). These results of the current study corroborate those of Chaiba et al. (2016) [9], who reported that 24% (18/75) of chicken farms were infected with *Salmonella* in Morocco. Moreover, our result was significantly higher than *Salmonella* chicken infection rate in China (11.38%; 33/290) (*p* < 0.05) indicated by Cui et al. (2016) [42]. In addition, our study revealed that the prevalence of *Salmonella* broiler infection was significantly higher during the hot season compared to the cold season (*p* < 0.05). High prevalence of infection during the hot season could be due to the high temperature and hygrometry, both favorable for *Salmonella* growth.

On the other hand, a total of 13 serotypes were identified in the current study. Five serotypes were predominant (*S*. Kentucky, *S.* Mbandaka, *S*. Anatum, *S*. Zanzibar, and *S.* Enteritidis) (*p* < 0.05) (Table 4). Our results show slight similarities with studies from other countries, such as Canada, the USA, Spain, and China, where *S.* Enteritidis and *S.* Typhimurium were the most prevalent [43]. In South Korea, Vietnam, and Cambodia, *S.* Hadar, *S.* Infantis, and *S.* Anatum were the most prevalent [44,45,46]. This suggests that there are geographical differences in the occurrence and dominance of *Salmonella* serotypes. In addition, the presence of *S*. Kentucky (20.3%; 13/64) revealed by our study represents a real threat to human health as it is often associated with multidrug resistance to several antimicrobial families, as indicated by Turki et al. (2012) [7].

Our study revealed that the presence of *Salmonella* spp. in broiler farm buildings is significantly favored by a lack of biosecurity standards, as well as a lack of compliance with animal welfare rules. Our results are in agreement with those of Chaiba et al. (2016) [9] and El Allaoui et al. (2017) [47], who found that the specific risk factors for *Salmonella* infection of broiler farms are linked to inadequate hygienic measures. Similarly, Heyndrickx et al. (2002) [48], Line et al. (2002) [49], and Cardinale et al. (2004) [50] indicated that chicken density greater than 25 subjects per square meter in broiler farm buildings, wet litter, and crawl space < 15 days are important risks of *Salmonella* broiler infection, respectively.

All studied *Salmonella* strains (64) were positive for the genes *invA, pagK, mgtC,* and *sirA,* and negative for the virulence genes *spvC, trhH, SEN1417, sipA, sipD,* and *sopD*. We found four virulotypes, namely, *invA-gipA-pagK-mgtC-sirA*, *invA-gipA-pagK-mgtC-sirA-Hli*, *invA-pagK-mgtC-sirA,* and *invA-pagK-mgtC-sirA-Hli*. The virulence genes could lead to serious cases of *Salmonella* foodborne infections in children, elderly, and immune-compromised persons.

The results of the present study are in agreement with those of Karraouan et al. (2010) [20] who reported that *Salmonella* (39 isolates) were positive for the *invA* gene in turkey meat in Morocco. In addition, most of the *Salmonella* serotypes isolated were negative for the *spvC* and *Hli* genes, while four strains (*S*. Kentucky) were positive for the *Hli* gene. The *spvC* gene was amplified only in a strain of *S.* Gallinarum. Moreover, Abouzeed et al. (2000) [27] amplified the invA gene in 75 *Salmonella* isolates of food and human origins. The results of the present study concerning the spvC gene differ from those of Abouzeed et al. (2000) [27]. These authors reported that 28% of *Salmonella* isolates were positive for the *spvC* gene (21/75). Our results agree with those of Turki et al. (2012) [7], who indicated that all strains (57) of *Salmonella* Kentucky isolated from different sources (animals, food, and human) were negative for the *spvC* gene.

As it was reported in several countries (France, Belgium, Slovak Republic, Morocco, and Ethiopia) [51,52,53], we noticed high resistance rates to nalidixic acid, amoxicillin, streptomycin, and ciprofloxacin, and a high multidrug-resistance (MDR) rate of 87.5% (56/64) in *Salmonella* isolated in the current study. Three MDR strains were extended-spectrum β-lactamase (ESBL)-producers, and three MDR strains were cephalosporinase-producers. Moreover, the antimicrobials often used in the visited poultry farms belong to the families of fluoroquinolones, beta-lactamines, second- and third-generation cephalosporin, sulfamides, and tetracycline. The high rates of resistance revealed by our study correspond to antimicrobials that belong to these families. Colistin is used very little in the visited poultry farms. It is considered the treatment of last resort prescribed for severe infections caused by bacteria resistant to commonly used antimicrobials. This therapeutic approach could explain the relatively low resistance rate to colistin.

The *bla_CTX-M_* gene was amplified in all the three ESBL strains. The *tetA*, *tetB,* and *dfrA1* genes were identified in a few resistant strains, but *qnrB* and *mrc* genes were not identified in any of the MDR *Salmonella* isolates. Genes of integrase class 2 were identified in 11% (7/64) of resistant *Salmonella* strains. Our results are comparable to those from the study of Lapierre et al. (2020) [54], who studied antimicrobial resistance of 87 *S*. Infantis isolates from chicken meat for sale in supermarkets in Santiago, Chile. In this study, high levels of multidrug-resistant and ESBL strains were indicated at the rate of 94 and 63%, respectively. The *bla_CTX-M-65_* gene was identified in 15% (13/87) of isolates. In addition, three isolates were resistant to fluoroquinolones, with the presence of the *qnrB* gene in two strains. The rate of resistance to colistin was high (29%), but the *mcr* genes were absent in the 87 studied strains. In addition, class 1 integrons were amplified in 7% (6/87) of the isolates [54].In the present study, some resistance genes were not detected in disc diffusion test positive isolates because resistance to an antimicrobial can be linked to different genes, but we cannot test all these genes.

An interesting result was reported in our study indicating high resistance rate to ertapenem. Carbapenem resistance, mainly among Gram-negative pathogens, is an ongoing public health problem of global dimensions. This type of antimicrobial resistance, especially when mediated by transferable carbapenemase-encoding genes, is spreading rapidly, causing serious outbreaks and dramatically limiting treatment options [55].In addition, the results of our study corroborate those of Ben Hassena et al. (2019) [56], who reported the emergence of multidrug-resistant *Salmonella* strains isolated from food with decreased susceptibility to fluoroquinolones and third-generation cephalosporin in Tunisia. Then, our findings show the importance of embracing the “One Health” approach that was promulgated by the World Health Organization in its action plan against antimicrobial resistance, which mobilizes various actors in three main sectors: animal husbandry, environment, and human health. In fact, the World Health Organization has named antimicrobial resistance as one of the three most important public health threats of the 21st century [57].

## 5. Conclusions

Twenty percent of the broiler flocks were infected with different serotypes of *Salmonella*. High resistance rates to nalidixic acid, amoxicillin, streptomycin, and ciprofloxacin were detected. An alarming level of resistance to ertapenem (12.5%) was noticed. Nearly ninety percent of strains were identified as multidrug-resistant (MDR). Extended-spectrum β-lactamases (ESBL)-producer and cephalosporinase-producer *Salmonella* strains were identified. The *bla_CTX-M_* gene was amplified in three ESBL strains. Therefore, vigorous control measures are needed in the first step of the poultry chain. In addition, the association of multidrug resistance and virulence genes in *Salmonella* isolates justifies the need for surveillance systems for both human and animal health sectors.

## Figures and Tables

**Table 1:** Functions and primers of *Salmonella* for virulence genes targeted in the present study.

Gene	Function	Primer Sequence (5′ to 3′)	Product Size (bp)	Annealing Temperature (°C)	Reference
*SEN1417*	Intracellular survival	F: GATCGCTGGCTGGTC	670	58	[22]
		R: CTGACCGTAATGGCGA			
*sipA*	Host cell invasion	F: ATGGTTACAAGTGTAAGGACTCAG	2055	53	[23]
		R: ACGCTGCATGTGCAAGCCATC			
*sipD*	Host cell invasion	F: ATGCTTAATATTCAAAATTATTCCG	1029	53	[23]
		R: TCCTTGCAGGAAGCTTTTG			
*sopD*	Host cell invasion	F: GAGCTCACGACCATTTGCGGCG	1291	59	[21]
		R: GAGCTCCGAGACACGCTTCTTCG			
*gipA*	Growth or survival in a Peyer’s patch	F: ACGACTGAGCAGGCTGAG	518	58	[25]
		R: TTGGAAATGGTGACGGTAGAC			
*mgtC*	Intracellular survival	F: TGACTATCAATGCTCCAGTGAAT	677	58	[25]
		R: ATTTACTGGCCGCTATGCTGTTG			
*trhH*	Code for the putative F pilus assembly protein	F: AACTGGTGCCGTTGTCATTG	418	53	[25]
		R: GATGGTCTGTGCTTGCTGAG			
*spvC*	Multiplication in host cell	F: CTCCTTGCACAACCAAATGCG	570	53	[25]
		R: TGTCTCTGCATTTCACCACCATC			
*sirA*	Control enteropathogenic virulence functions	F: TGCGCCTGGTGACAAAACTG	313	55	[25]
		R: ACTGACTTCCCAGGCTACAGCA			
*pagK*	Biofilm formation	F: ACCATCTTCACTATATTCTGCTC	151	60	[25]
		R: ACCTCTACACATTTTAAACCAATC			
*invA*	Host cell invasion	F: GTGAAATTATCGCCACGTTCGGGCAA	284	64	[26]
		R: TCATCGCACCGTCAAAGGAACC			
*Hli*	Control of phase change and motility	F: AGCCTCGGCTACTGGTCTTG	173	55	[27]
		R: CCGCAGCAAGAGTCACCTCA			

F: Forward primer; R: Reverse primer.

**Table 2:** Primers of *Salmonella* for antimicrobial resistance genes targeted in the present study.

Gene	Primer Sequence (5′ to 3′)	Product Size (bp)	Annealing Temperature (°C)	Reference
*bla_TEM_*	F: ATCAGCAATAAACCAGC	516	54	[30]
	R: CCCCGAAGAACGTTTTC			
*bla_CTX-M_*	F: ATGTGCAGYACCAGTAARGTKATGGC	592	58	[31]
	R: TGGGTRAARTARGTSACCAGAAYSAGCGG			
*bla_NDM1_*	F: CTGAGCACCGCATTAGCC	621	52	[33]
	R: GGGCCGTATGAGTGATTGC			
*tetA*	F: GGTTCACTCGAACGACGTCA	577	55	[34]
	R: CTGTCCGACAAGTTGCATGA			
*tetB*	F: CCTCAGCTTCTCAACGCGTG	634	55	[34]
	R: GCACCTTGCTGATGACTCTT			
*dfrA1*	F: GGAGTGCCAAAGGTGAACAGC	367	55	[35]
	R: GAGGCGAAGTCTTGGGTAAAAAC			
*qnrB*	F: GATCGTGAAAGCCAGAAAGG	469	53	[32]
	R: ACGATGCCTGGTAGTTGTCC			
*mcr-1*	F: AGTCCGTTTGTTCTTGTGGC	320	58	[36]
	R: AGATCCTTGGTCTCGGCTTG			
*mcr-2*	F: CAAGTGTGTTGGTCGCAGTT	715	58	[36]
	R: TCTAGCCCGACAAGCATACC			
*mcr-3*	F: AAATAAAAATTGTTCCGCTTATG	929	58	[36]
	R: AATGGAGATCCCCGTTTTT			
*mcr-4*	F: TCACTTTCATCACTGCGTTG	1116	58	[36]
	R: TTGGTCCATGACTACCAATG			
*mcr-5*	F: ATGCGGTTGTCTGCATTTATC	1644	58	[36]
	R: TCATTGTGGTTGTCCTTTTCTG			
*int1*	F: GGGTCAAGGATCTGGATTTCG	483	62	[37]
	R: ACATGGGTGTAAATCATCGTC			
*int2*	F: CACGGATATGCGACAAAAAGGT	233	62	[37]
	R: GTAGCAAACGAGTGACGAAATG			

F: Forward primer; R: Reverse primer.

**Table 3:** Univariate and multivariate analysis of *Salmonella* prevalence in broiler according to the studied risk factors.

Risk Factor	Category	Prevalence in % (Positive/Tested)	OR [95% CI]	*p*-Value	Multivariate Logistic Regression OR [95% CI]
No cleaning and disinfection around the breeding unit	Yes	29.4 (40/136)	2.810 [1.600–4.950]	**<0.001**	**8.642 [1.770–42.196]**
	No	12.9 (24/186)			
Absence of treatment with an antimicrobial at the start	Yes	33.3 (52/156)	6.420 [3.270–12.610]	**<0.001**	**4.675 [1.720–12.703]**
	No	7.2 (12/166)			
Duration of crawl space < 15 days	Yes	32.7 (53/162)	6.590 [3.290–13.200]	**<0.001**	**3.562 [1.436–8.835]**
	No	6.9 (11/160)			
Wet litter	Yes	27.8 (50/180)	3.520 [1.850–6.680]	**<0.001**	
	No	9.9 (14/142)			
Hot season (T ≥ 20 °C) *	Yes	24.6 (55/224)	3.218 [1.520–6.813]	**0.001**	
	No	9.2 (9/98)			
Number of chicks at setting in place > 25/m^2^	Yes	26.6 (47/177)	2.720 [1.480–4.990]	**0.009**	
	No	11.7 (17/145)			
Absence of rodent control in the building	Yes	17.6 (42/238)	0.604 [0.335–1.089]	0.092	
	No	26.2 (22/84)			
Poor state of cleanliness of poultry	Yes	18.7 (45/241)	0.749 [0.408–1.375]	0.351	
	No	23.5 (19/81)			

OR: odds ratio, CI: confidence interval, *p* < 0.05: variable significantly associated with infection with *Salmonella* spp. In bolded characters: significant *p* value and multivariate logistic regression OR. (*): Hot season (May–October) characterized by an average ambient temperature ≥ 20 °C.

**Table 4:** *Salmonella* serotypes and virulence genes isolated in the present study (n = 64).

Serotypes Prevalence in % (Positive/Tested)	Strains	Virulence Genes ^(a)^											
		*invA*	*spvC*	*hli*	*gipA*	*mgtC*	*trhH*	*sirA*	*pagK*	*sipA*	*sipD*	*sopD*	*SEN*
*S.* Kentucky 20.3% (13/64)	E2	+	−	−	−	+	−	+	+	−	−	−	−
	E8	+	−	+	+	+	−	+	+	−	−	−	−
	E11	+	−	−	+	+	−	+	+	−	−	−	−
	E17	+	−	−	−	+	−	+	+	−	−	−	−
	E22	+	−	+	+	+	−	+	+	−	−	−	−
	E24	+	−	+	+	+	−	+	+	−	−	−	−
	E25	+	−	−	−	+	−	+	+	−	−	−	−
	E31	+	−	+	+	+	−	+	+	−	−	−	−
	E36	+	−	−	+	+	−	+	+	−	−	−	−
	E38	+	−	−	+	+	−	+	+	−	−	−	−
	E40	+	−	+	−	+	−	+	+	−	−	−	−
	F1	+	−	+	+	+	−	+	+	−	−	−	−
	F4	+	−	+	−	+	−	+	+	−	−	−	−
*S.* Mbandaka 18.7% (12/64)	E12	+	−	−	+	+	−	+	+	−	−	−	−
	E16	+	−	−	+	+	−	+	+	−	−	−	−
	E18	+	−	+	+	+	−	+	+	−	−	−	−
	E20	+	−	+	+	+	−	+	+	−	−	−	−
	E23	+	−	−	+	+	−	+	+	−	−	−	−
	E32	+	−	−	+	+	−	+	+	−	−	−	−
	E39	+	−	−	+	+	−	+	+	−	−	−	−
	M23	+	−	−	−	+	−	+	+	−	−	−	−
	M25	+	−	−	+	+	−	+	+	−	−	−	−
	M29	+	−	+	−	+	−	+	+	−	−	−	−
	M34	+	−	+	+	+	−	+	+	−	−	−	−
	F6	+	−	−	+	+	−	+	+	−	−	−	−
*S.* Anatum 17.1% (11/64)	M17	+	−	−	+	+	−	+	+	−	−	−	−
	M19	+	−	−	+	+	−	+	+	−	−	−	−
	M21	+	−	−	+	+	−	+	+	−	−	−	−
	M22	+	−	+	+	+	−	+	+	−	−	−	−
	M24	+	−	+	+	+	−	+	+	−	−	−	−
	M26	+	−	−	−	+	−	+	+	−	−	−	−
	M28	+	−	+	+	+	−	+	+	−	−	−	−
	M30	+	−	+	+	+	−	+	+	−	−	−	−
	M31	+	−	−	+	+	−	+	+	−	−	−	−
	M32	+	−	−	−	+	−	+	+	−	−	−	−
	M33	+	−	−	−	+	−	+	+	−	−	−	−
*S.* Zanzibar 15.6% (10/64)	E3	+	−	−	+	+	−	+	+	−	−	−	−
	E5	+	−	−	+	+	−	+	+	−	−	−	−
	E6	+	−	+	+	+	−	+	+	−	−	−	−
	E7	+	−	−	+	+	−	+	+	−	−	−	−
	E26	+	−	−	+	+	−	+	+	−	−	−	−
	E28	+	−	−	+	+	−	+	+	−	−	−	−
	E29	+	−	−	+	+	−	+	+	−	−	−	−
	E34	+	−	+	+	+	−	+	+	−	−	−	−
	E35	+	−	−	+	+	−	+	+	−	−	−	−
	F3	+	−	−	+	+	−	+	+	−	−	−	−
*S.* Enteritidis 12.5% (8/64)	E1	+	−	−	+	+	−	+	+	−	−	−	−
	E13	+	−	−	+	+	−	+	+	−	−	−	−
	E14	+	−	−	+	+	−	+	+	−	−	−	−
	E15	+	−	+	+	+	−	+	+	−	−	−	−
	E21	+	−	+	+	+	−	+	+	−	−	−	−
	E27	+	−	−	−	+	−	+	+	−	−	−	−
	E30	+	−	−	+	+	−	+	+	−	−	−	−
	E33	+	−	−	+	+	−	+	+	−	−	−	−
*S.* Infantis 3.1% (2/64)	M27	+	−	−	+	+	−	+	+	−	−	−	−
	F2	+	−	−	+	+	−	+	+	−	−	−	−
*S.* Indiana 3.1% (2/64)	E10	+	−	−	+	+	−	+	+	−	−	−	−
	M20	+	−	+	+	+	−	+	+	−	−	−	−
*S.* Corvallis 1.6% (1/64)	E9	+	−	−	+	+	−	+	+	−	−	−	−
*S.* Agona 1.6% (1/64)	E4	+	−	−	+	+	−	+	+	−	−	−	−
*S.* Hadar 1.6% (1/64)	E19	+	−	+	+	+	−	+	+	−	−	−	−
*S.* Montevideo 1.6% (1/64)	M18	+	−	−	−	+	−	+	+	−	−	−	−
*S.* Cerro 1.6% (1/64)	F5	+	−	+	+	+	−	+	+	−	−	−	−
*S.* Virginia 1.6% (1/64)	E37	+	−	−	+	+	−	+	+	−	−	−	−

^(a)^ +: Present; −: Absent.

**Table 5:** *Salmonella* serotypes and antimicrobial resistance profiles isolated in the present study (n = 64).

Serotypes Prevalence in % (Positive/Tested)	Strains	Antimicrobial Resistance Profiles ^**(b)**^																				
		AMX	AUC	SF	FOX	CAZ	CTX	CRO	CPM	ATM	ETP	GME	S	CS50	NA	ENF	CIP	FFC	C	TE	SUL	SXT
*S.* Kentucky 20.3% (13/64)	E2 ^**(c)**^																					
	E8 ^**(c)**^																					
	E11 ^**(c)**^																					
	E17 ^**(c)**^																					
	E22 ^**(c)**^																					
	E24 ^**(c)**^																					
	E25 ^**(c)**^																					
	E31 ^**(c)**^																					
	E36 ^**(c)**^																					
	E38 ^**(c)**^																					
	E40 ^**(c)**^																					
	F1 ^**(c)**^																					
	F4 ^**(c)**^																					
*S.* Mbandaka 18.7% (12/64)	E12 ^**(c)**^																					
	E16 ^**(c)**^																					
	E18 ^**(c)**^																					
	E20 ^**(c)**^																					
	E23 ^**(c)**^																					
	E32 ^**(c)**^																					
	E39 ^**(c)**^																					
	M23 ^**(c)**^																					
	M25 ^**(c)**^																					
	M29 ^**(c)**^																					
	M34 ^**(c)**^																					
	F6 ^**(f)**^																					
*S.* Anatum 17.1% (11/64)	M17 ^**(d)**^																					
	M19 ^**(e)**^																					
	M21 ^**(e)**^																					
	M22 ^**(d)**^																					
	M24 ^**(f)**^																					
	M26 ^**(d)**^																					
	M28 ^**(f)**^																					
	M30 ^**(c)**^																					
	M31 ^**(c)**^																					
	M32 ^**(c)**^																					
	M33 ^**(c)**^																					
*S.* Zanzibar 15.6% (10/64)	E3 ^**(c)**^																					
	E5 ^**(c)**^																					
	E6 ^**(c)**^																					
	E7 ^**(c)**^																					
	E26 ^**(c)**^																					
	E28 ^**(c)**^																					
	E29 ^**(c)**^																					
	E34 ^**(c)**^																					
	E35 ^**(c)**^																					
	F3 ^**(c)**^																					
*S.* Enteritidis 12.5% (8/64)	E1 ^**(c)**^																					
	E13 ^**(c)**^																					
	E14 ^**(f)**^																					
	E15 ^**(c)**^																					
	E21 ^**(c)**^																					
	E27 ^**(c)**^																					
	E30 ^**(c)**^																					
	E33 ^**(c)**^																					
*S.* Infantis 3.1% (2/64)	M27 ^**(c)**^																					
	F2 ^**(c)**^																					
*S.* Indiana 3.1% (2/64)	E10 ^**(c)**^																					
	M20 ^**(c)**^																					
*S.* Corvallis 1.6% (1/64)	E9 ^**(e)**^																					
*S.* Agona 1.6% (1/64)	E4 ^**(f)**^																					
*S.* Hadar 1.6% (1/64)	E19 ^**(c)**^																					
*S.* Montevideo 1.6% (1/64)	M18 ^**(f)**^																					
*S.* Cerro 1.6% (1/64)	F5 ^**(f)**^																					
*S.* Virginia 1.6% (1/64)	E37 ^**(f)**^																					

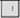
**^(b)^** Sensitive; 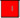 Resistant. AMX: amoxicillin, AUC: amoxicillin + clavulanic acid, SF: cefalotin, FOX: cefoxitin, CAZ: ceftazidim, CTX: cefotaxim, CRO: ceftriaxon, CPM: cefepim, ATM: aztreonam, ETP: ertapenem, GME: gentamicin, S: streptomycin, CS50: colistin, NA: nalidixic acid, ENF: enrofloxacin, CIP: ciprofloxacin, FFC: florfenicol, C: chloramphenicol, TE: tetracycline, SUL: sulfamides, SXT: trimethoprim-sulfamethoxazole. **^(c)^** MDR^+^ Strain, **^(d)^** MDR^+^ & ESBL^+^ Strain, **^(e)^** MDR^+^ & AmpC^+^ Strain, MDR^−^, **^(f)^** ESBL^−^ & AmpC^−^ Strain.

## Data Availability

The study did not report any data.
